# Transcriptome analysis for *Caenorhabditis elegans *based on novel expressed sequence tags

**DOI:** 10.1186/1741-7007-6-30

**Published:** 2008-07-08

**Authors:** Heesun Shin, Martin Hirst, Matthew N Bainbridge, Vincent Magrini, Elaine Mardis, Donald G Moerman, Marco A Marra, David L Baillie, Steven JM Jones

**Affiliations:** 1 Molecular Biology and Biochemistry, Simon Fraser University, Burnaby, BC, Canada; 2 Canada's Michael Smith Genome Sciences Centre, British Columbia Cancer Research Centre, British Columbia Cancer Agency, Vancouver, BC, Canada; 3 Genome Sequencing Center, Washington University School of Medicine, St Louis, USA; 4 Department of Zoology, University of British Columbia, Vancouver, BC, Canada

## Abstract

**Background:**

We have applied a high-throughput pyrosequencing technology for transcriptome profiling of *Caenorhabditis elegans *in its first larval stage. Using this approach, we have generated a large amount of data for expressed sequence tags, which provides an opportunity for the discovery of putative novel transcripts and alternative splice variants that could be developmentally specific to the first larval stage. This work also demonstrates the successful and efficient application of a next generation sequencing methodology.

**Results:**

We have generated over 30 million bases of novel expressed sequence tags from first larval stage worms utilizing high-throughput sequencing technology. We have shown that approximately 14% of the newly sequenced expressed sequence tags map completely or partially to genomic regions where there are no annotated genes or splice variants and therefore, imply that these are novel genetic structures. Expressed sequence tags, which map to intergenic (around 1000) and intronic regions (around 580), may represent novel transcribed regions, such as unannotated or unrecognized small protein-coding or non-protein-coding genes or splice variants. Expressed sequence tags, which map across intron-exon boundaries (around 300), indicate possible alternative splice sites, while expressed sequence tags, which map near the ends of known transcripts (around 600), suggest extension of the coding or untranslated regions. We have also discovered that intergenic and intronic expressed sequence tags, which are well conserved across different nematode species, are likely to represent non-coding RNAs. Lastly, we have incorporated available serial analysis of gene expression data generated from first larval stage worms, in order to predict novel transcripts that might be specifically or predominantly expressed in the first larval stage.

**Conclusion:**

We have demonstrated the use of a high-throughput sequencing methodology to efficiently produce a snap-shot of transcriptional activities occurring in the first larval stage of *C. elegans *development. Such application of this new sequencing technique allows for high-throughput, genome-wide experimental verification of known and novel transcripts. This study provides a more complete *C. elegans *transcriptome profile and, furthermore, gives insight into the evolutionary and biological complexity of this organism.

## Background

Computationally based genomic analyses have been able to accomplish interpretation of the genome of *Caenorhabditis elegans *on a global scale. The aims of some high-throughput genomic projects have focused on the identification of developmental stage, tissue or cell-specific 'transcriptomes', which attempt to describe transcribed regions and their relative abundance [1-3].

Approaches such as microarray, serial analysis of gene expression (SAGE) [4], and expressed sequence tag (EST) analysis have been widely used for the identification of genes that are selectively turned on or off in specific cell or tissue types with regard to development, aging, and disease. These approaches have also provided experimental evidence for the confirmation of predicted gene structures, alternative splice variants [5], and non-coding RNAs (ncRNAs) [6].

At present, there are approximately 340,000 *C. elegans *ESTs in WormBase which, in addition to available cDNA sequences, provide complete transcriptional evidence for 34.6% of the transcripts. The remaining transcripts are partially confirmed by ESTs or only computationally predicted by comparative genomics or *ab-initio *gene prediction methods (WS180 release notes).

We have sequenced a large number of ESTs from a *C. elegans *cDNA population, synchronized at the first larval (L1) developmental stage, by a high-throughput, sequencing-by-synthesis technology, namely 454 sequencing [7]. This method produces DNA sequences more rapidly and cost-effectively than the traditional Sanger sequencing approach and has been successfully utilized in other studies for various purposes, such as expression profiling and novel gene discovery [8-10]. We have generated more than 300,000 novel *C. elegans *EST sequences by this highly parallel sequencing method for this study.

We have analyzed the novel sequence data to obtain a more complete *C. elegans *transcriptome profile, providing not only confirmation of computationally predicted transcripts but also the identification of potential novel transcripts, alternatively spliced variants, and ncRNAs [11]. In addition, the increased depth of this sequencing of *C. elegans *L1 cDNA library facilitated a more sensitive search for novel transcribed regions that may be specific for the first larval stage of *C. elegans*. We have also investigated conservation of potential novel transcribed regions across available nematode species namely: *C. elegans, C. briggsae*, *C. remanei, C. brenneri, Brugia malayi *and *Pristionchus pacificus*.

## Results and discussion

### 454 EST sequencing identifies known transcripts and partially confirms computationally predicted transcripts

Using sequencing-by-synthesis technology we have generated a total of 300,453 reads (30,907,940 bases) from an L1-specific cDNA sample with an average read length of 102 bases. An average 454 read accuracy is measured to be 99.4% with substantially all of the bases having Phred 20 or better quality [7]. Sequences identified as vector contamination were filtered out using Crossmatch [12], resulting in a data set of 298,838 454 ESTs, which were aligned using the Basic Local and Alignment and Search Tool (BLAST) [32], to around 22,000 known and predicted *C. elegans *genes (WormBase release WS160). From this set, a total of 229,989 454 ESTs (77%) were directly mapped to 6132 known or predicted *C. elegans *genes by BLAST with high confidence value (*P*-value less than 9 × 10^-7^). Transcripts which have the greatest number of 454 ESTs (250 to 10,000), generally match ribosomal protein coding genes. This is expected as ribosomal protein coding genes are the most abundantly expressed type of genes. These data provide partial experimental evidence for approximately 200 genes, which have previously been predicted only through computational methods (Additional file 1).

Around 22% of the 454 EST data (66,358 reads) had no significant matches to known or predicted *C. elegans *transcripts at the specified stringency and as such may represent previously unidentified genetic structures, such as novel transcripts, L1 stage-specific transcripts, novel splice variants and ncRNAs. The remaining 1% (2491 reads) of the data ambiguously map to more than one transcript at the high stringency used, although these ambiguous matches are usually simple repeats or sequences of low-complexity.

### 454 EST reads are biased towards 3'-transcript ends

**Figure 1:**
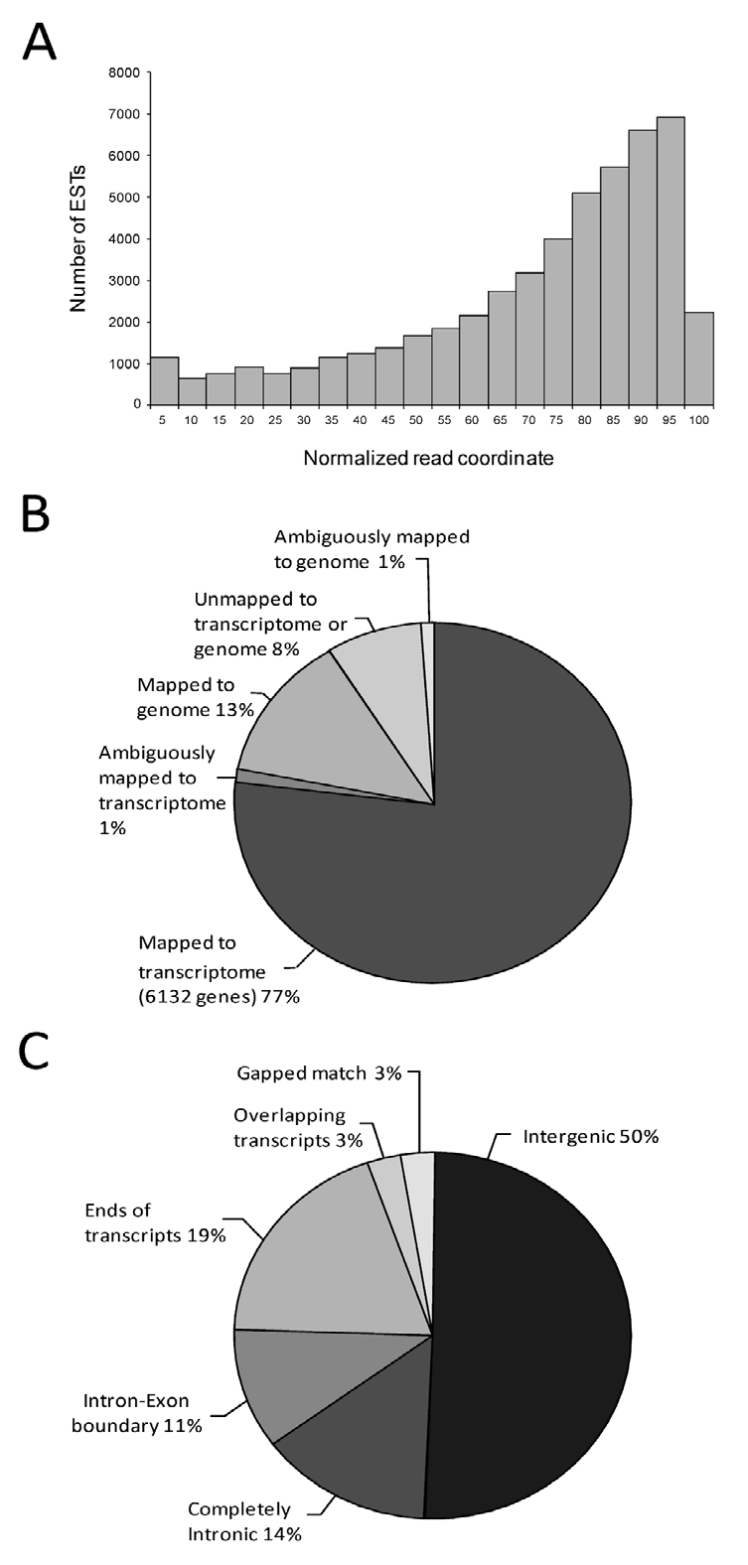
**454 ESTs mapping to *Caenorhabditis elegans *transcripts**. (A) Histogram showing the distribution of 454 expressed sequence tags (ESTs) mapping to *Caenorhabditis elegans *transcripts. Coordinate 0 on the *x*-axis represents the 5'-end of the transcripts. (B) Summary of 454 EST mapping result to the *C. elegans *transcriptome and genome. (C) Categorization of genomic 454 EST hits.

The physical distribution of 454 EST reads, which map across known transcripts from their 5'- to 3'-ends, shows a larger coverage on the 3'-ends (Figure 1A). This is likely due to the presence of partial transcripts in the cDNA library. The lack of splice leader sequences also indicates under-representation of the 5'-ends of the transcripts. On average, six unique EST reads were mapped to each known transcript ranging from a single EST to 847 unique ESTs, with a median of two ESTs.

### Most statistically over-represented genes identified by 454 ESTs correlate to developmental, reproductive, and cellular metabolic processes

We performed gene ontology (GO) analysis on 6132 genes identified by 454 ESTs using GOstat [14]. The most statistically over-represented GO annotations in biological processes (*P*-value less than 9 × 10^-10^) within this group of genes correlate to multicellular organismal development processes (that is, larval development, post-embryonic body morphogenesis, positive regulation of growth, and homeostatic process), reproductive developmental processes in a multicellular organism (that is, sex differentiation, gamete generation, genitalia development, and oviposition), and lastly, cellular metabolic processes (that is, translation, cellular component organization and biogenesis, co-enzyme biosynthetic process, and protein and RNA metabolic processes).

### 454 ESTs that map to *C. elegans' *genome identify putative novel transcripts or splice variants

The 22% (66,358 reads) of 454 ESTs, which did not have significant matches to known or predicted *C. elegans *transcripts, was subsequently compared with the genomic sequence of *C. elegans *using BLAST. As a result, 31,570 ESTs (14%) map to the genome at a high stringency (that is, *P*-value less than 9 × 10^-5^). A stringent *P*-value threshold of 9 × 10^-7 ^was used for mapping 454 ESTs to the transcriptome to ensure that read alignments to the transcriptome were of very high quality and unlikely to occur by chance. Subsequently the less stringent threshold of 9 × 10^-5 ^was used here for alignments against the genome. Although this increases the chance of incorrect alignment, it increases the total number of aligned reads and may facilitate the discovery of novel transcription events, which can subsequently be validated.

The remaining ESTs (8%), which do not map to either the transcriptome or genome are composed of contamination, low complexity or poor quality sequences (Figure 1B). From this analysis, 530 additional genes (along with 6132 genes found in the previous step) were identified by ESTs mapping completely to their introns or partially to the exons.

### Genomic EST hits are categorized according to genomic mapping locations

The 31,570 ESTs that align to the genome have been subdivided into the following categories: ESTs which map to intergenic regions (50%), intronic regions (14%), and transcript ends and/or untranslated regions (UTRs) (19%), EST reads that split into two separate alignment blocks (3%), and those which span exon and intron boundaries suggesting alternative splice junctions (11%). The last 3% mapped to overlapping transcripts (Figure 1C). We have investigated each category to search for genetic structures, such as putative novel genes, splice variants, and ncRNA genes from each genomic region. Some of the genomic regions already have computational predictions or other previously sequenced ESTs supporting the presence of such structures, and some do not have any other information to support these findings. Lack of other experimental and computational evidence may indicate possible splicing errors or unknown genetic features.

### Intergenic ESTs

We have classified intergenic ESTs as those ESTs which mapped within intergenic regions of the genome but did not overlap with adjacent genes. A total of 8449 intergenic ESTs mapped to 1061 intergenic regions ranging from single counts to coverage with over 1000 reads (including identical ESTs); 120 of these intergenic regions have five or more unique ESTs mapping within them (Additional file 2).

Most intergenic regions (around 850) have one unique EST cluster (that is, identical and overlapping ESTs) mapped, around 150 intergenic regions have two clusters, around 35 intergenic regions have three clusters, around 20 have four clusters, and, finally, one intergenic region has 14 EST predicted clusters.

**Figure 2:**
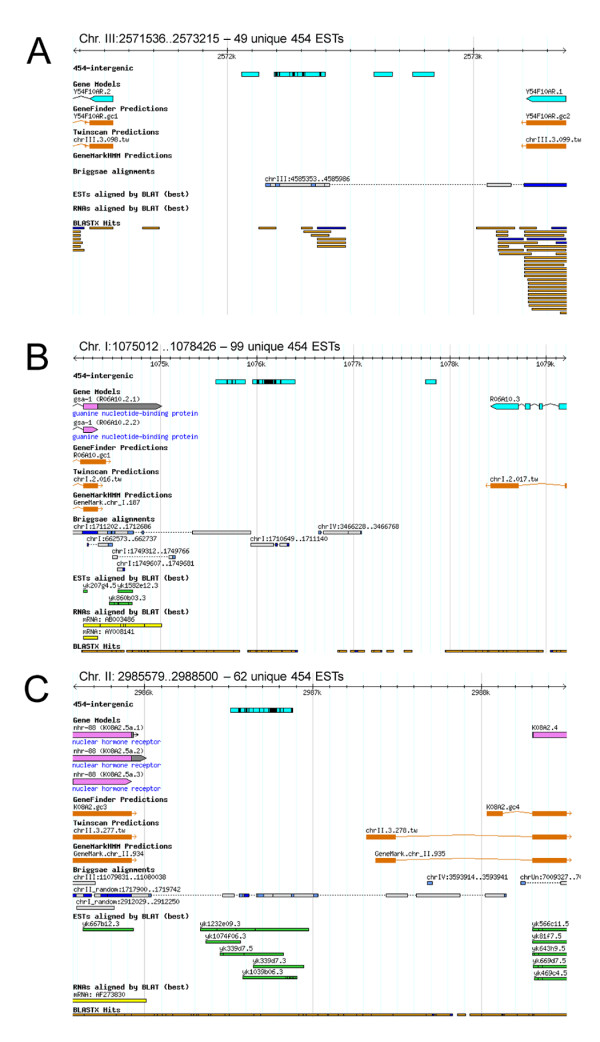
**The intergenic region on chromosomes with unique 454 expressed sequence tags**. (A) The intergenic region on chromosome III with 49 unique 454 expressed sequence tags (ESTs). (B) The intergenic region on chromosome I with the most number of unique 454 ESTs (99) in this analysis. (C) The intergenic region on chromosome II with 62 unique 454 ESTs. The 454 EST clusters in the middle of these intergenic regions with black vertical bars represent deep EST coverage, and conservation of these regions between *Caenorhabditis elegans *and *C. briggsae *is shown. These ESTs may represent a novel gene or extension of the neighboring gene. Note that the genomic regions shown are not to the same scale.

Figure 2 shows the intergenic regions with the most ESTs. There are no protein-coding gene predictions in these intergenic regions, although there are some small open reading frames within these intergenic regions. However, as indicated in the Figure 2, the regions where most ESTs map show a high conservation between *C. elegans *and *C. briggsae*. In addition, BLAST analysis of these regions (nucleotide to protein via six-frame translation) reveals protein homology against reference protein data sets from the genomes of yeast, fly, worm, and human, and also against SwissProt and TREMBL [15]. These EST loci may represent novel genes that are small or extensions of neighboring genes.

Interestingly, the neighboring gene to the novel EST hits shown in Figure 2B, *gsa-1 *(R06A10.2), encodes a Gs alpha subunit of heterotrimeric G proteins, which affects L1 stage viability, movement, and egg laying [16]. *gsa-1 *is confirmed both by previously sequenced cDNAs and ESTs. We postulate that the 454 ESTs may, therefore, indicate a UTR extension of *gsa-1 *or, alternatively, a splice variant. It is also interesting to note that the number of 454 ESTs mapped near the 3'-end of *gsa-1 *is much greater than the number of previously sequenced ESTs mapped to *gsa-1*. This may indicate that 454 EST sequencing has much deeper coverage of L1 stage mRNA sample or, alternatively, the potential novel splice variant shows a relatively higher level of expression at the L1 stage.

Finally, previously sequenced ESTs (Yuji Kohara, unpublished) overlap with 454 ESTs in the intergenic region shown in Figure 2C. These ESTs support a possible 3'-end extension of the neighboring gene, *nhr-88 *(K08A2.5a). The ESTs generated by Yuji Kohara (yk1039b06, yk1074f06, yk1232e09), are also generated from an L1 stage *C. elegans *cDNA library. As *nhr-88 *has been determined to belong to a gene cluster containing genes that are significantly enriched in L1 muscle [17], this example implies that our 454 EST sequencing data has deep coverage of L1-enriched genes.

The size of the intergenic regions to which the 454 ESTs map ranges from 114 base pairs (bp) to 38,046 bp. Although we observe a positive correlation between the physical distribution of EST hits and the intergenic size (Pearson correlation coefficient of 0.46), we found no correlation between the size of the intergenic region and the number of ESTs that mapped to it. The distribution of intergenic EST hits across intergenic regions was observed to be relatively uniform, which is unexpected given that we anticipated witnessing a bias towards the ends of the intergenic regions (that is, close to neighboring genes), which would likely represent UTRs or novel terminating or initiating exons of the bordering genes. EST hits in the middle of large intergenic regions distant from neighboring genes represent more likely candidates for novel transcripts, including ncRNA genes [18].

### Intronic ESTs

**Figure 3:**
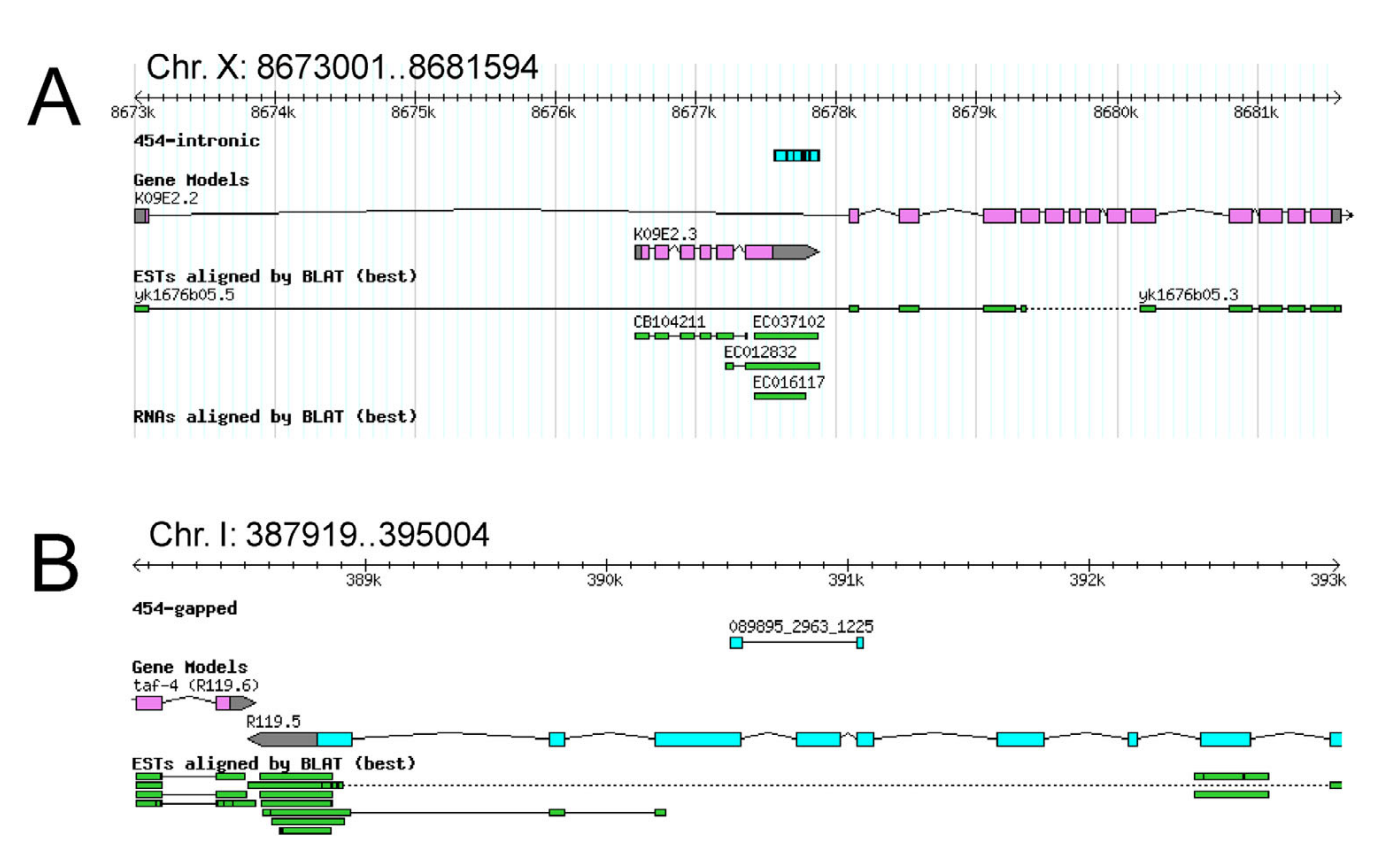
**Examples of intronic and gapped expressed sequence tags**. (A) An example of intronic expressed sequence tags (ESTs) showing 454 ESTs mapped to the gene, *K09E2.3*, which is added to a recent WormBase release (WS180) within the intron of *K09E2.2*. There are also other ESTs recently added that confirm *K09E2.3*. (B) An example of a gapped-EST suggesting alternative splicing or correction of the current gene model. Note that the genomic regions shown are not to the same scale.

We have classified intronic ESTs as those ESTs which mapped completely within introns. Intronic EST matches may represent novel exons (that is, alternative splicing), as well as novel overlapping transcripts on the opposite strand. ncRNAs are also known to be present in intronic regions [11]. A total of 1921 ESTs with over 90% alignment were mapped within introns of 584 *C. elegans *transcripts using BLAST (*P*-value less than 9 × 10^-5^); see Additional file 3. Of these genes, 262 only had the intronic EST hits without any ESTs completely mapping to their annotated exons. These ESTs may indicate that there are ncRNA genes or novel transcripts on the opposite strand within the introns. The reasoning behind this speculation is that the probability of a gene having only intronic ESTs without any ESTs mapping to its annotated exons is low for the possibility that the intronic ESTs are derived from a novel exon of that gene. In fact, the recent WormBase version (WS180) added four new protein coding genes within some of these introns but on the opposite DNA strand and the ESTs mapped in the introns match those new genes (Figure 3A and Additional file 4).

### 5'- and 3'-end ESTs

5'- and 3'-end ESTs are ESTs that partially map to the beginning or end of transcripts (that is, 5'- and 3'-UTRs or terminating/initiating exons). These EST matches are also interesting in that they may contain regulatory elements, such as subcellular localization signals [19], and *cis*-elements for mRNA stability and translation [20]. We found 131 transcripts with ESTs mapping to their 5'-ends (Additional file 5) and 956 transcripts had ESTs mapped to their 3'-ends. These 3'- and 5'-end 454 ESTs represent UTRs or coding region extensions potentially including alternative start and stop codons.

### Gapped ESTs

When one end of an EST read maps to a genomic location and the other end of the EST read maps to a location some distance away (that is, two separate alignment blocks), we have categorized these as 'gapped ESTs'. ESTs that map to the ends of two adjacent exons confirming known introns (approximately 29,000 ESTs) are not included in this data set as they were examined in the initial comparison to the transcriptome. Fifteen such 'gapped EST' hits were found (Figure 3B), confirming novel exon-intron boundaries and providing strong experimental evidence for novel transcripts or alternative splice variants with skipped exons, novel internal or end exons, or novel exon-intron boundaries (Table 1). Six of these EST matches confirm updated gene structures in recent WormBase release (WS180).

**Table 1:** Summary of gapped-expressed sequence tag matches and putative novel structures

**% Cov**	**Gap size**	**Chromosome**	**First alignment block**	**Second alignment block**	**Putative novel structure**
98	480	I	390514 to 390558	391038 to 391059	Skipped exon
96	489	I	5761023 to 5761058	5761547 to 5761592	Novel end exon confirmed*
98	101	I	7642453 to 7642487	7642588 to 7642631	Novel end exon
98	62	I	9724862 to 9724908	9724970 to 9725025	Alternate exon-intron boundary
98	157	I	11931105 to 11931163	11931320 to 11931345	Novel internal exon
92	45	II	2782924 to 2782962	2783007 to 2783086	Novel end exon confirmed*
98	224	II	10828328 to 10828404	10828628 to 10828667	Confirmed intron*
91	461	II	13635576 to 13635653	13636114 to 13636163	Confirmed intron*
98	548	II	14747166 to 14747225	14747773 to 14747821	Novel end exon
98	306	III	8552492 to 8552538	8552844 to 8552868	Alternate exon-intron boundary
92	277	III	11751557 to 11751606	11751883 to 11751921	Novel end exons confirmed*
92	1364	III	12639534 to 12639558	12640922 to 12640967	Novel internal exon
98	926	IV	9350535 to 9350565	9351491 to 9351529	Confirmed intron*
97	900	V	1981345 to 1981373	1982273 to 1982364	Novel end exons
98	50	X	11824129 to 11824180	11824230 to 11824259	Novel transcript/novel end exons

* A recent WormBase release (WS180) has since confirmed these exon-intron boundaries; the original mapping analysis was performed using WB160.

### Exon-intron boundary ESTs

ESTs that map across exon and intron boundaries are a possible indication of novel alternative splicing events (that is, alternative 5'- or 3'-end splice sites) or, alternatively, cDNAs that have been partially processed with some introns left intact. These EST hits could also provide experimental confirmation for incorrect splice site predictions in the current gene models, particularly for those that lack experimental validation. Additional file 6 lists 284 transcripts with 454 ESTs that map across their exon and intron boundaries.

### Exon-intron boundaries with 454 ESTs mapped show weaker 3'-end splice site conservation

**Figure 4:**
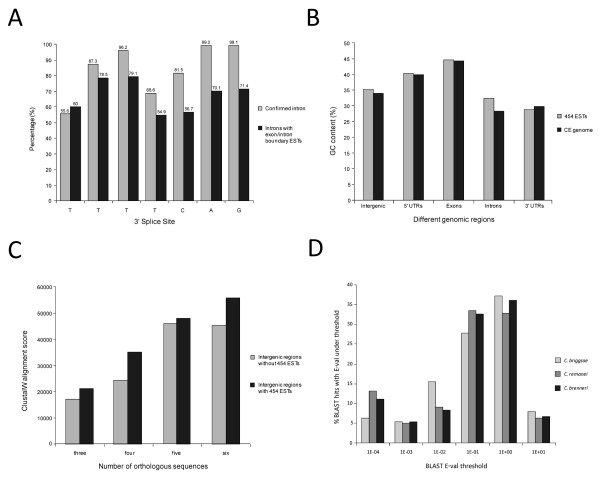
**Comparative analyses of 3' splice sites, GC contents, and cross-species sequence conservation**. (A) The conservation of consensus 3'-end splice sites (TTTTCAG) of confirmed transcripts and transcripts with exon-intron boundary 454 expressed sequence tag hits. (B) A comparison of 454 expressed sequence tags and *Caenorhabditis elegans *whole genome for guanine-cytosine content of different genomic regions. (C) Average ClustalW alignment score comparisons for intergenic regions with or without 454 expressed sequence tags for different numbers of orthologous sequences. (D) Chart showing 3524 unique *Caenorhabditis elegans *intergenic 454 expressed sequence tags mapped to *C. briggsae, C. remanei*, and *C. brenneri*.

We have analyzed splice sites for the transcripts which have ESTs mapped across exon-intron boundaries, and found the consensus 3'-end splice sites (that is, TTTTCAG) are less well conserved compared with the transcripts that have splice sites confirmed by ESTs or RNAs as shown in Figure 4A. The weaker conservation of the 3'-end splice sites may be a feature of alternative splice sites or simply be more prone to erroneous splicing events.

### 454 ESTs mapped to different genomic regions show average guanine-cytosine contents similar to the genomic averages

Comparisons of guanine-cytosine (GC) contents of the 454 EST read sequences, in different genomic regions, with overall *C. elegans *genomic sequences are shown in Figure 4B. As expected, EST read sequences that mapped to exons have the highest GC content, and these are close to the average for annotated coding sequences of the whole genome (around 45%). It might be expected that 454 EST reads, which mapped to intergenic and intronic regions, would have GC content close to that of the coding sequence if they represent novel transcripts or exons. However, the intergenic and intronic EST read sequences have similar percentage GC to the average GC contents of intergenic and intronic sequences for genome, around 34% and 28%, respectively, suggesting the ESTs that map to intergenic regions or introns represent evidence for non-coding, transcribed sequences rather than protein coding sequences as ncRNA genes do not show as strong base composition biases as do protein coding sequences [21].

### Intergenic regions with 454 ESTs show a higher degree of cross-species conservation

Conservation of intergenic regions across different nematode species is evidence for functional genetic structures. These intergenic regions, with 454 ESTs mapped to them, were aligned with other nematode species namely: *C. briggsae*, *C. remanei*, *C. brenneri*, *B. malayi*, and *P. pacificus*, using ClustalW. Approximately 1000 intergenic regions in total were randomly selected for this analysis, providing a 1:1 ratio of intergenic regions with 454 ESTs aligned to and ones without. These intergenic regions had three to six orthologous sequences for multiple sequence alignments depending on the availability and existence of orthologous sequences. Intergenic regions with mapped 454 ESTs had overall higher average ClustalW alignment scores than the ones without 454 ESTs (Figure 4C). This higher degree of conservation of the intergenic regions with 454 ESTs represents further evidence that supports the presence of putative novel functional transcripts identified by the 454 EST sequences.

### Cross-species EST-to-genome comparisons identify highly conserved ESTs and species-specific ESTs

Another important analysis is the cross-species comparison of ESTs that map to the genome. We have compared the well-annotated genomes of *C. elegans *and *C. briggsae*, as well as the *C. remanei *genome that has more recently become available.

Strong and abundant EST matches on well-conserved genomic regions is strong evidence supporting the presence of novel genetic structures. We were interested in comparing EST hits and cross-species conservation of the genomic regions where the ESTs align. Such characterization of EST hits unique to one species and EST hits in conserved regions may offer evolutionary clues to alternative splicing.

We have examined both species-specific and species-conserved splicing events by mapping the intergenic 454 EST sequences to *C. briggsae*, *C. remanei*, and *C. brenneri *by BLAST. A total of 3524 unique *C. elegans *ESTs, which were aligned to intergenic regions at the high stringency, were mapped to *C. briggsae*, *C. remanei*, and *C. brenneri*. The top 5% of the BLAST hits, with the highest scores, were most common among the three nematodes, but *C. remanei *had the greatest number of high score BLAST hits (around 15%) with *E*-values lower than 1 × 10^-4 ^(Figure 4D). The intergenic regions where these ESTs are aligned are highly conserved across species as expected, and synteny of these genomic regions also seems to be well conserved (data not shown). These highly conserved EST hits across species likely represent novel transcripts. EST alignments with poor scores, such as BLAST hits with an *E*-value higher than 10, indicate that the ESTs mapped uniquely to *C. elegans *at high stringency. These EST sequences may be from novel transcripts that are unique to *C. elegans*, although it is also possible that some or all of these ESTs may be transcriptional noise.

### Highly conserved ESTs are mapped to ncRNAs

ncRNAs are anticipated to be conserved [13]. The conservation of primary structure for ncRNAs is known to be variable when the secondary structure is expected to be more highly conserved across species [22]. It is also known that expression of ncRNAs vary with developmental stages [23], and therefore, our ESTs may identify ncRNAs highly expressed in the L1 stage.

**Figure 5:**
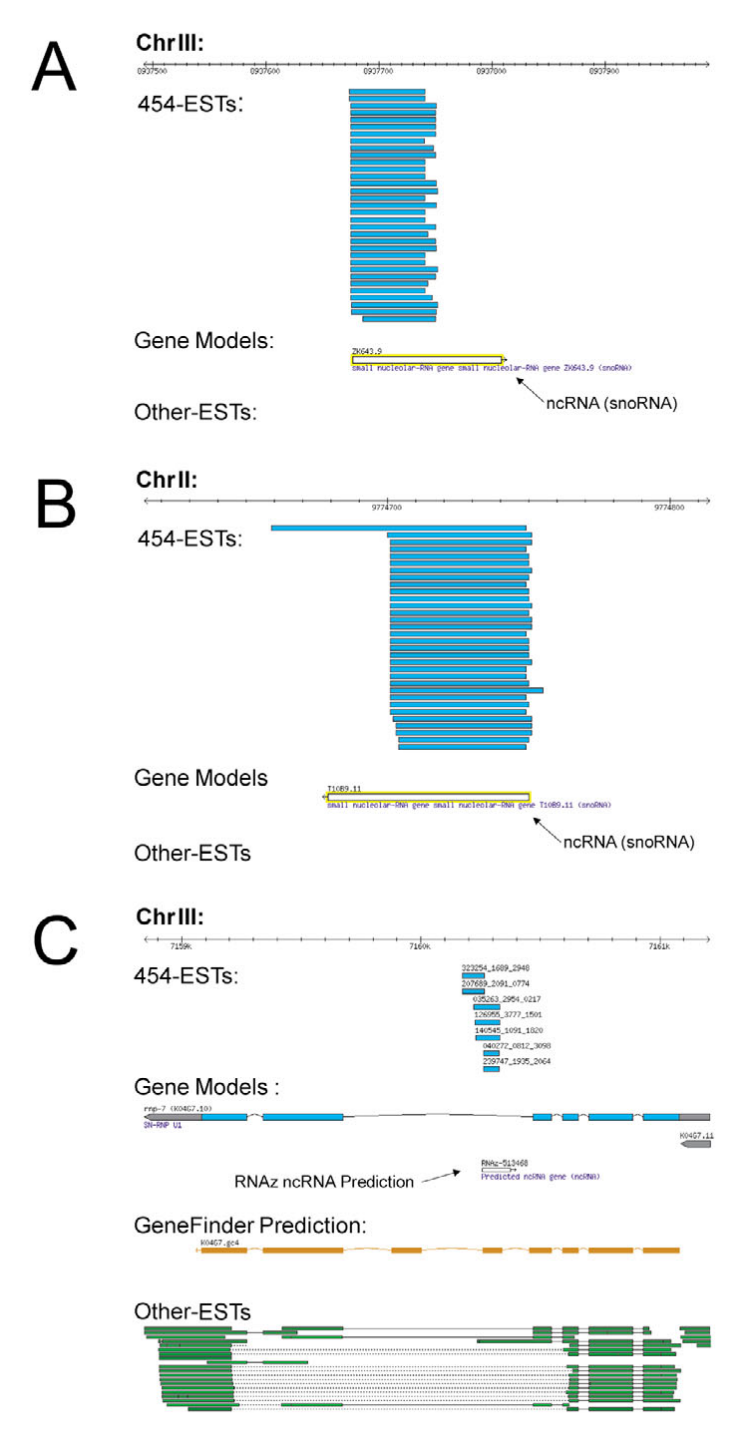
**Examples of 454 ESTs mapped to known or predicted ncRNAs**. (A), (B) Representative 454 expressed sequence tag data, which identify known non-coding RNAs. (C) The most conserved cross-species intronic 454 expressed sequence tags hit mapping to a RNAz non-coding RNA prediction. Note that the genomic regions shown are not to the same scale.

We have selected and examined the EST loci that are highly conserved across species (*E*-values lower than 1 × 10^-4^), and have at least five or more EST reads mapped in the middle of large intergenic regions (more than 10 kb) away from neighboring genes (Table 2). These EST hits are the most probable candidates for novel transcripts and in fact, many of these EST loci are either mapped to ncRNAs that are identified, confirmed and added to more recent WormBase or ncRNA predictions performed by RNAz [24] (Figure 5A and 5B).

**Table 2:** Most highly conserved 454 expressed sequence tags loci in intergenic regions

**Count**	**Intergenic coordinates**	**Intergenic distance**	**Distance to nearest gene**	**Expressed sequence tags count**
1	I:12824792..12825228	436	10	6
2	I:1841201..1842444	1243	107	13
3	II:11162247..11171658	9411	4258	5
4	II:15226621..15230280	3659	763	13
5	II:5956644..5961678	5034	612	16
6	II:9773798..9774904	1106	150	9
7	III:11170439..11171970	1531	377	17
8	III:12130215..12152775	22560	7576	5
9	III:1733092..1743238	10146	4249	10
10	III:1743309..1752172	8863	1819	6
11	III:2768655..2770841	2186	687	6
12	III:3664472..3667602	3130	79	19
13	III:4098100..4121436	23336	1088	8
14	III:8935324..8944721	9397	1136	18
15	IV:9934182..9935039	857	229	25
16	V:1075619..1081451	5832	939	18
17	V:1361139..1373418	12279	403	5
18	V:1980421..1982526	2105	6	11
19	V:5932735..5933473	738	180	5
20	V:6336709..6357288	20579	1448	6
21	X:15012843..15026225	13382	4845	8
22	X:15616440..15620416	3976	928	7
23	X:16927937..16944791	16854	1211	22
24	X:837757..838677	920	315	6

The most conserved intronic ESTs map to *C. elegans *gene *K04G7.10 *and its *C. briggsae *ortholog. Consistent with well conserved EST loci in intergenic regions, these ESTs may represent an ncRNA as they overlap with an ncRNA prediction within the intron, however, alternatively this could be a novel exon as GeneFinder has predicted an exon in that region (Figure 5C).

### 454 ESTs support computational ncRNA gene predictions

Currently, there are around 1300 annotated functional ncRNAs in WormBase [11], of which 39 are in our data set, including snoRNAs, miRNAs, 21URNAs, rRNAs, and some ncRNAs that could not be assigned to any functional class (Table 3). We suspect that 454 ESTs may represent precursor RNAs, such as pre-miRNA and pre-snoRNAs, which are known to be polyadenylated [25-27], since our RNA preparation was done using a polyA-dependent method. However, it is also possible that potential ncRNAs that are identified in this study may belong to polyadenylated ncRNA classes, such as mRNA-like ncRNAs (mlncRNAs) [28].

**Table 3:** Non-coding RNA genes identified by 454 expressed sequence tags (incomplete)

**RNA type**	**Gene name**	**Chromosome**	**Status**	**Location**
miRNA	Y105E8A.31	I	Predicted	3'-UTR of Y105E8A.16
miRNA	F08F3.11	V	Predicted	Intergenic
Non-coding RNA	F54D7.7	I	Predicted	Overlap with 3'-UTR of F54D7.4
21URNA	C46G7.7	IV	RNAs	Intergenic
21URNA	C08F11.38	IV	RNAs	Intron of C08F11.13
21URNA	T23G4.18	IV	RNAs	Intergenic
21URNA	T23G4.24	IV	RNAs	Intergenic
21URNA	F55B11.13	IV	RNAs	Intergenic
21URNA	Y105C5A.159	IV	RNAs	Intergenic
21URNA	Y51H4A.106	IV	RNAs	Intergenic
Non-coding RNA	F09E10.10	X	Expressed sequence tags	Intergenic
Non-coding RNA	C30E1.9	X	Expressed sequence tags	Intergenic
RNA pseudogene	D1005.t1	X	Predicted	Intergenic
RNA pseudogene	ZK380.t2	X	Expressed sequence tags	Intergenic
rRNA	F31C3.11	I	Expressed sequence tags	Intergenic
rRNA	F31C3.9	I	Expressed sequence tags	Intergenic
snoRNA	R12E2.17	I	RNAs	Intron of R12E2.3
snoRNA	F25H5.9	I	RNAs	Intron of F25H5.3
snoRNA	T10B9.11	II	RNAs	Intergenic
snoRNA	M106.6	II	RNAs	Intron of M106.1
snoRNA	H06I04.9	III	Predicted	Intron of H06I04.4
snoRNA	ZK643.9	III	RNAs	Intergenic
snoRNA	Y43B11AR.7	IV	Expressed sequence tags	Intron of Y43B11AR.4
snoRNA	F17C11.14	V	RNAs	Intron of F17C11.9
snoRNA	K09E9.5	X	mRNA	Intergenic

Single-sequence RNA secondary structure predictions, without using comparative genomes, only take into account thermodynamic models and energy minimization, which are not sufficient to achieve the necessary sensitivity and specificity for ncRNA prediction. For that reason, we have compared the 454 EST mapping result with RNAz ncRNA predictions [24], which incorporate homology information of the RNA secondary structure to make predictions for ncRNAs. This approach, using *C. elegans *and *C. briggsae*, has proven fruitful in identifying over 2000 putative RNA loci [18].

We compared RNAz ncRNA predictions for *C. elegans *and 332 unique 454 ESTs mapped to intergenic regions. We found 19 ncRNA predictions in close proximity to 454 ESTs (within 100 bp), with nine of these predicted ncRNAs overlapping with the 454 ESTs in intergenic regions (Table 4). We have also compared the intronic 454 ESTs with the RNAz ncRNA predictions and found that 10 introns contained both 454 ESTs and ncRNA predictions within 100 bp, and five introns had intronic ESTs that overlap with ncRNA predictions (Table 5). These ESTs that map to ncRNA predictions may represent novel ncRNAs.

**Table 4:** Intergenic 454 expressed sequence tags overlapping with RNAz non-coding RNA predictions

**Expressed sequence tags**	**Expressed sequence tags coordinate**	**Predicted non-coding RNA coordinate**	**Intergenic coordinate**
062385_1158_0389	I:9863279..9863316	I:9863183..9863302	I:9862287..9863587
004771_1566_3251	II:15165861..15165910	II:15165800..15165950	II:15165619..15166104
074784_3939_2069	III:11474420..11474530	III:11474520..11474625	III:11474411..11479792
313519_0405_2992	IV:3168808..3168913	IV:3168772..3168891	IV:3155983..3169942
320686_1504_2153	V:5440646..5440778	V:5440615..5440736	V:5438973..5446748
322033_3470_0718	V:11801636..11801764	V:11801572..11801690	V:11800350..11803726
240717_0095_3913	V:12296851..12296944	V:12296911..12297022	V:12293608..12302290
093179_3480_2283	X:17010136..17010216	X:17010094..17010200	X:17008275..17013376
104650_3438_2367	X:6961371..6961474	X:6961430..6961548	X:6956349..6967668

**Table 5:** Intronic 454 expressed sequence tags overlapping with RNAz non-coding RNA predictions

**Expressed sequence tag coordinate**	**Predicted non-coding RNA coordinate**	**Intron coordinate**	**Gene**
III:4689667..4689728	III:4689566..4689715	III:4689399..4690021	*T04A8.5*
III:7160222..7160329	III:7160256..7160375	III:7159674..7160467	*K04G7.10*
V:18041446..18041591	V:18041383..18041481	V:18041071..18041867	*Y59A8B.6*
V:18041446..18041593	V:18041501..18041624	V:18041071..18041867	*Y59A8B.6*
V:6881222..6881352	V:6881154..6881264	V:6881209..6881399	*K11C4.3*
X:9091394..9091461	X:9091460..9091597	X:9090452..9092219	*H08J11.2*

### L1 SAGE and 454 ESTs overlap by 50%

We have investigated both the commonalities and differences between the large amount of available SAGE data [3,29] and the novel 454 EST data. Both data sets were generated from the same mRNA preparation of L1 stage animals and as such are a direct comparison of these two gene expression-measuring techniques.

The number of genes that were identified by SAGE and ESTs independently from L1 stage animals is 5115 and 6132, respectively, but the number of genes that were identified by both methods is only 3068. Hence, 2047 genes were identified by SAGE only and 3064 genes were identified only by ESTs. It is worth noting that while the same mRNA sample was used for both SAGE and 454 EST analyses, the inherent differences in the technologies used may have introduced discrepancies in gene identification. For example, approximately half of the genes identified by SAGE only have a single SAGE tag, which may not be sufficient evidence for expression of those genes due to the possibility of erroneous assignment [4], and approximately 12% of the genes identified only by 454 ESTs do not contain *NlaIII *restriction enzyme site required for a transcript to be identified by SAGE [4,30].

Spearman correlation of the transcript abundances, measured by SAGE and ESTs, was calculated using genes that have both SAGE tags and ESTs mapped to them. The correlation coefficient is 0.36, which is not as high as we initially expected considering both EST and SAGE libraries were prepared from the same mRNA sample. This, however, raises interesting questions as to how well each data set represents the complete picture of transcriptional activities. It could be that from the large scale of transcriptional activities, each snap-shot represents only a partial picture, or that each experiment contains significant amounts of new information, although it could simply be due to discrepancies between different gene expression profiling methods.

### L1 SAGE and 454 ESTs identify putative novel L1 stage-enriched genes

We have compared 454 ESTs and SAGE tags, which map specifically to intergenic regions. There are 166 intergenic regions that have both L1 454 ESTs and L1 SAGE tags mapped to them (Additional file 7). When we examined intergenic regions with SAGE tags, which are enriched in the L1 stage but lowly expressed in embryo and other developmental stages, we observed a good correlation between the L1-enriched SAGE tags and L1 454 ESTs. In other words, most intergenic regions with SAGE tags that are expressed highly in the L1 stage also had 454 ESTs mapped in close proximity. We speculate these loci may represent novel coding or non-coding transcripts that are potentially L1 stage specific but expressed in low abundance. Additionally, we postulate that both intergenic SAGE tags enriched in L1 and L1 454 ESTs, which map together in regions without any genes in their vicinity may represent putative novel transcripts that may be enriched in the L1 stage of *C. elegans*. In addition, ESTs and SAGE tags which map near the 3'-end of genes might represent 3'-UTR extensions and as such can provide evidence of expression for those genes at the L1 stage.

## Conclusion

We have successfully demonstrated the use of the next-generation sequencing technology (454 sequencing-by-synthesis approach) for transcriptome analysis in an extremely efficient manner. We have identified a number of putative novel genetic structures from the transcriptome snap-shot obtained from this analysis, including putative novel splice variants and ncRNAs that might be stage specific.

## Methods

### mRNA and cDNA preparation

Total RNA from a pooled sample was prepared using TRIZOL Reagent (Invitrogen Life Technologies, Carlsbad, CA) following the manufacturer's instructions and was assayed for quality and quantified using an Agilent 2100 Bioanalyzer (Agilent Technologies, Mississauga, ON) and RNA 6000 Nano LabChip kit (Caliper Technologies, Hopkinton, MA). Contaminating genomic DNA was removed from total RNA by DNAse1 treatment using DNAfree (Ambion, Austin, TX), following the manufacturer's instructions. First-strand cDNA synthesis was prepared from 2 μg of total RNA using the Powerscript Reverse Transcriptase (cat#639501, Takara Bio Inc. Shiga, Japan). For the first-strand synthesis, custom biotinylated primers containing a recognition sequence for the type IIS restriction enzyme Mme1 were used at a final concentration of 1 μM (454-3F-biotin, 5'-/Biotin/-AAG CAG TGG TAA CAA CGC ATC CGA CTT TTT TTT TTT TTT TTT TTT TTV N-3'; 454-3A, AAG CAG TGG TAA CAA CGC AGA GTA CGC GGG). The resulting single-stranded cDNA was amplified using the Advantage 2 polymerase chain reaction (PCR) kit (BD Biosciences Clontech, Mountain View, CA) in a final volume of 1000 μl with the addition of 454-3A at a final concentration of 240 nM. The cycling conditions consisted of an initial denaturation at 95°C for 1 minute followed by 20 cycles of 95°C for 30 seconds, 65°C for 30 seconds and 68°C for 6 minutes. After amplification, the DNA was recovered using a QIAquick PCR Purification kit (Qiagen) according to manufacturer's instructions. Following column elution, the DNA was bound to pre-washed M280 Streptavidin beads (Dynal Biotech) and subjected to Mme1 digestion according to manufacturer's instructions (New England Biolabs) in the presence of S-adenosylmethionine. Following a 2.5 hour incubation at 37°C, the supernatant was removed and subjected to phenol chloroform isoamyl alcohol (pH 8.0, 100 μl; Fisher) extraction in phase-lock gel tubes (heavy) 0.5 ml (Eppendorf) and the 600 μl aqueous phase precipitated by the addition of 2750 μl of 100% ethanol, 8 μl of mussel glycogen (Invitrogen), and 360 μl of 7.5 M ammonium acetate, and incubation at -20°C overnight. The precipitate was recovered by centrifugation at 14,000 rpm for 30 minutes at 4°C in an Eppendorf benchtop refrigerated centrifuge (model 5810R) and washed in 70% ethanol, resuspended in 14 μl dH_2_O. The DNA quality was assessed and quantified using an Agilent DNA 1000 series II assay (Agilent). In preparation for 454 sequencing, 3 μg of the cDNA sample was nebulized to a mean fragment size of 600 ± 50 bp, end-repaired and adapter-ligated according to the standard procedures described previously [7].

### 454 sequencing and sequence analysis

We adapted the standard procedures for 454 sequencing described previously [7]. We also followed standard post-run, bioinformatics processing on the 454 platform to determine reads that passed various quality filters.

After high quality sequence reads were obtained, BLAST analysis was performed as in Bainbridge et al [2]. Sequences were first trimmed of low quality bases using trim2 (-M 10) [31] and mapped to *C. elegans *transcriptome (WormBase release WS160) using wuBLAST (version 2.0, 10 May 2005) [32]. BLAST hits with a *P*-value of 9 × 10^-7 ^or less (comparable to the BLAST *E*-value of around 9 × 10^-13^), which corresponds approximately to a 60-bp contiguous perfect match in the data set, were considered to be successful hits against the transcriptome. Sequences that did not map to *C. elegans *transcriptome were then aligned with wuBLAST to *C. elegans *genome (*P*-value of 9 × 10^-5 ^or less, comparable to the BLAST *E*-value of around 9 × 10^-11^). The positions of significant hits with respect to exons, introns, intergenic regions, ESTs, SAGE tags and other DNA alignment features were determined using the Perl Ensembl API (version 35) and Ensembl database (WormBaseWS160). Also, ClustalW (version 1.74) was used for cross-species, multiple sequence alignments.

## List of abbreviations

BLAST: Basic Local Alignment Search Tool; bp: base pairs; EST: expressed sequence tag; GC: guanine-cytosine; GO: gene ontology; L1: first larval; ncRNA: non-coding RNA; PCR: polymerase chain reaction; SAGE: serial analysis of gene expression; UTR: untranslated region.

## Authors' contributions

DLB, MAM and SJMJ conceived of the study. HS performed the analyses and drafted the manuscript. DGM provided mRNAs. MH prepared cDNA libraries and wrote the corresponding methods section. VM and EM carried out DNA sequencing. MNB designed the mapping algorithm.

## Supplementary Material

Additional file 1 Computationally predicted genes (*WS170*) partially confirmed by 454 expressed sequence tags. Click here for file

Additional file 2 Intergenic regions with five or more unique 454 expressed sequence tags. Click here for file

Additional file 3 List of genes with 454 expressed sequence tags mapped within their introns. Click here for file

Additional file 4 Genes with five or more intronic 454 expressed sequence tags only. *A more recent WormBase (WS180) added new genes within the introns where these expressed sequence tags map Click here for file

Additional file 5 Genes with 5'-end 454 expressed sequence tags with over 90% alignment. Click here for file

Additional file 6 List of genes with exon-intron boundary 454 expressed sequence tags. Click here for file

Additional file 7 166 intergenic regions with both first larval stage 454 expressed sequence tags and first larval stage SAGE. Click here for file
